# Factors Influencing Health-Related Quality of Life of Patients with Spinocerebellar Ataxia

**DOI:** 10.1007/s12311-024-01657-2

**Published:** 2024-01-27

**Authors:** Niklas Weber, Maresa Buchholz, Anika Rädke, Jennifer Faber, Tanja Schmitz-Hübsch, Heike Jacobi, Thomas Klockgether, Wolfgang Hoffmann, Bernhard Michalowsky, the EUROSCA study group, Sophie Tezenas du Montcel, Peter Bauer, Paola Giunti, Arron Cook, Robyn Labrum, Michael H. Parkinson, Alexandra Durr, Alexis Brice, Perrine Charles, Cecilia Marelli, Caterina Mariotti, Lorenzo Nanetti, Marta Panzeri, Maria Rakowicz, Anna Sulek, Anna Sobanska, Ludger Schöls, Holger Hengel, Laszlo Baliko, Bela Melegh, Alessandro Filla, Antonella Antenora, Jon Infante, José Berciano, Bart P. van de Warrenburg, Dagmar Timmann, Sandra Szymanski, Sylvia Boesch, Jun-Suk Kang, Massimo Pandolfo, Jörg B. Schulz, Sonia Molho, Alhassane Diallo, the ESMI study group, Jeanette Hübener-Schmid, Magda Santana, Marcus Grobe-Einsler, Berkan Koyak, Mafalda Raposo, Manuela Lima, Hector Garcia-Moreno, Paola Giunti, Luís Pereira de Almeida, Bart van de Warrenburg, Judith van Gaalen, Dagmar Timmann, Andreas Thieme, Kathrin Reetz, Imis Dogan, Carlo Wilke, Ludger Schöls, Olaf Riess, Matthis Synofzik, Jeroen de Vries, Jon Infante, Oz Gulin, James Joers, Chiadikaobi Onyike, Michal Povazan, Eva-Maria Ratai, Jeremy Schmahmann

**Affiliations:** 1 German Center for Neurodegenerative Diseases e.V. (DZNE), Patient-Reported Outcomes and Health Economics Research, Site Rostock/Greifswald, Ellernholzstraße 1-2, 17487 Greifswald, Germany; 2 German Center for Neurodegenerative Diseases e.V. (DZNE), Bonn, Germany; 3 Department of Neurology, University Hospital Bonn, Bonn, Germany; 4 Neuroscience Clinical Research Center (NCRC), Charité - Universitätsmedizin Berlin, Berlin, Germany; 5 Department of Neurology, University Hospital Heidelberg, Heidelberg, Germany; 6 Institute for Community Medicine, University Medicine Greifswald, Greifswald, Germany

**Keywords:** Spinocerebellar ataxia, Quality of life, mental health, EQ-5D

## Abstract

**Background:**

Little is known about the progression of health-related quality of life (HRQoL) and predicting factors in spinocerebellar ataxia (SCA). Such knowledge is crucial to identify modifiable factors promoting everyday life with SCA and attenuating HRQoL decline.

**Objectives:**

This study is to assess HRQoL progression and identify factors affecting SCA patients’ HRQoL.

**Methods:**

Longitudinal data (three-year follow-up) of 310 SCA patients of the European SCA3/Machado-Joseph-Disease Initiative (ESMI) (2016-2022) and 525 SCA patients (SCA1, SCA2, SCA3 or SCA6) of the EUROSCA natural history study cohort (2006–2015) were assessed. Both large cohort studies share standardized assessments of clinical measures, SARA, INAS, PHQ-9, and HRQoL (EQ-5D-3L). The association between HRQoL and clinical measures was assessed by Spearman Correlation (r^s^). Multivariable panel regression models were performed to evaluate the impact of patients’ socio-demographics, age of onset, SCA type and body mass index (BMI), and clinical measures on HRQoL progression.

**Results:**

HRQoL significantly decreased over one (− 0.014, *p* = 0.095), two (− 0.028, *p* = 0.003), and three years (− 0.032, *p* = 0.002). Ataxia severity and mental health strongly correlated with HRQoL (r^s^_SARA_ = − 0.589; r^s^_PHQ-9_ = − 0.507). HRQoL more intensively declined in male (ß = − 0.024, *p* = 0.038) patients with an earlier age of onset (ß = 0.002, *p* = 0.058). Higher progression of ataxia severity (ß = − 0.010, *p* ≤ 0.001), mental health problems (ß = − 0.012, *p* < 0.001), and higher BMI (ß = − 0.003, *p* = 0.029) caused more severe decline of patients’ HRQoL over time.

**Discussion:**

In absence of curative treatments, stronger focus on mental health and weight influence could help clinical evaluation and accompany treatment improving SCA patients’ HRQoL, especially in male patients with early disease onset.

**Supplementary Information:**

The online version contains supplementary material available at 10.1007/s12311-024-01657-2.

## Introduction

Spinocerebellar ataxias (SCAs) are autosomal-dominant inherited, progressive movement disorders comprising more than 40 genetically identified SCAs [1, 2]. The most common are SCA1, SCA2, SCA3, and SCA6, which comprise about 50% of all affected families. SCAs share an onset in adult life that can differ significantly among the SCA types [3]. SCA1, SCA2, and SCA3 have an average onset at around 40 years, whereas SCA6 has an average onset at 60 years and slower progression [2, 4].

Depending on the disease progression, almost all patients with SCA are increasingly limited in their everyday lives and suffer a shorter life expectancy [5, 6]. The clinical progression of SCAs is characterized by ongoing ataxia with imbalance, coordination deficits and slurred speech, increasing non-ataxia symptoms like sensory loss and muscle weakness, and cognitive and mental health problems [7, 8]. The long-term progression of clinical outcomes in patients with SCA was evaluated in two previous studies [5, 9], demonstrating that symptoms such as depression or fatigue increased throughout the disease. Schmitz-Hübsch et al. studied associated factors of self-rated health in SCA patients in a cross-sectional study sample [10]. The study conducted a multivariate analysis, indicating that subjective health rated on a visual analogue scale (EQ VAS) in SCA patients was associated with ataxia severity, non-cerebellar involvement, and the presence of depressive health status. So far, there is no curative treatment available and therapeutic approaches focus on managing the symptoms of SCAs [5]. Thus, it is important to understand the progression and factors that might influence patients’ well-being.

The physical limitations and mental health burden caused by SCA diseases can directly affect the patients’ health-related quality of life (HRQoL) [11]. HRQoL describes the individual’s perceived physical and mental health status and well-being [12] and has become crucial to understand, compare and predict outcomes of patients, and support shared decision-making in the treatment process [13]. Furthermore, better HRQoL has been found to be a prognostic factor for better overall survival of patients with various chronic illnesses [14]. Furthermore, Jacobi et al. looked at patient-reported outcomes in SCA patients longitudinally by using the EQ VAS instrument to identify associations and looking at EQ-5D dimensions’ progressions separately [15]. The findings showed that self-rated health is associated with ataxia severity and dysphagia in SCA1. However, the study could not show any associated factors with self-rated health in SCA2, SCA3, and SCA6.

So far, no generic HRQoL measure has been evaluated longitudinally in SCA patients apart from the use of the EQ VAS as a visual analogue scale. The SF-36 and EQ-5D-3L indexes were used in the cross-sectional analysis for SCA3 patients [16]. Additionally, SF-36 and the EQ-5D-3L index have been analysed cross-sectionally for SCA10 [17] and SCA12 patients [16]. Therefore, like with all chronic diseases, knowledge about modifiable factors affecting patients’ HRQoL is vital to slow down disease progression and improve long-term outcomes.

So far, only Jacobi et al. have looked at factors that might influence self-rated health [15]. However, studies that use longitudinal data to analyse the long-term progression of HRQoL in SCA patients are lacking. Thus, this study aimed to demonstrate the progression of HRQoL over time and assess factors influencing HRQoL in SCA1, SCA2, SCA3, and SCA6 patients.

## Methods

### Study Design, Recruitment, and Sample

We used data from two longitudinal, prospective, observational SCA cohort studies: (1) the European Spinocerebellar Ataxia Registry (EUROSCA), initiated in 2005 and followed up patients with SCA1, SCA2, SCA3, and SCA6 for 11 years in 17 European study centers [6, 17], and (2) the European Spinocerebellar Ataxia Type 3/Machado-Joseph Disease Initiative (ESMI), which started in 2016 as a multicentre cohort study of SCA3 patients, their first-degree relatives, and healthy controls [18]. Patients with SCA3 were recruited for ESMI from 11 European and three US study centers [18]. Written informed consent was obtained from all study participants before enrolment in both studies. The EUROSCA study is registered at ClinicalTrials.gov, number NCT02440763. Ethical committees of the participating centres approved the EUROSCA and ESMI study [6, 18].

### Sample Selection

**Fig. 1:**
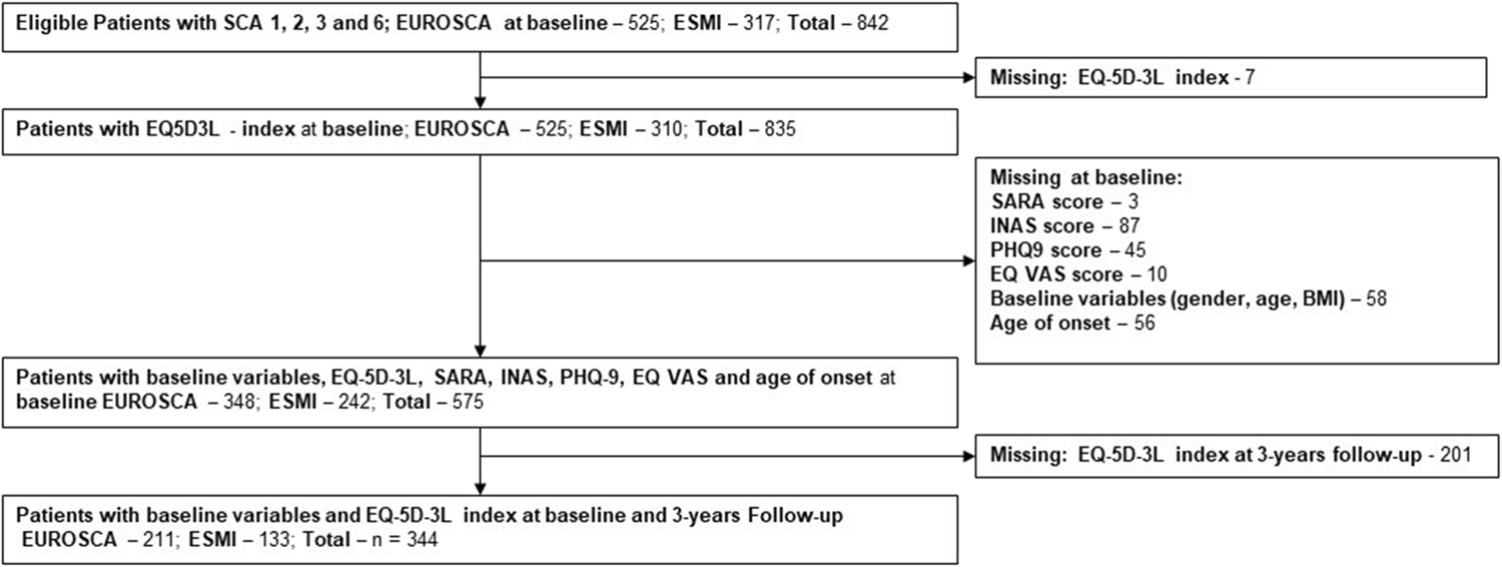
Flow chart for sample selection with imputed data from patient sample at baseline to 3-year-Follow-up time point

In total, 1140 participants gave informed consent and partook in the studies (EUROSCA, 677 participants; ESMI, 463 participants). Of these, 298 were healthy controls (EUROSCA, 152 participants; ESMI, 146 participants) excluded from this analysis. Thus, 842 patients with manifest SCA (EUROSCA, 525; ESMI, 310) were included at baseline. Until the 3-year follow-up, standardized study site consultations were scheduled yearly with a permitted deviation of two months. After the 3-year follow-up, study centre consultations could be organized more freely. To improve the quality and robustness of the results, we solely used data between baseline and 3-year follow-up without missing baseline data. The flow chart in Fig. 1 illustrates the participant flow, explaining inclusion and exclusion of participants.

### Data Assessment and Measures

In both studies, patients completed a standardized array of questionnaires and socio-demographics (age, gender, age of ataxia onset) at baseline and each yearly follow-up [17, 18]. For baseline assessment, age was categorized age into age groups. Patients’ body mass index (BMI) was assessed and categorized in WHO criteria as underweight (< 18.5 kg/m^2^), normal weight (18.5–24.9 kg/m^2^), overweight (25–29.9 kg/m^2^), obese I (30–34.9 kg/m^2^), obese II (35–39.9 kg/m^2^), and obese III (> 40 kg/m^2^) [19]. Restless legs syndrome (RLS) diagnosis was present in both datasets. An RLS variable was created to reduce disparities between the diagnosis in both datasets. Furthermore, we included SCA1, SCA2, SCA3, and SCA6 patients in the data sets; however, we introduced a dichotomous variable, SCA type, to account for the distinction between the more similar SCA1–SCA3 and SCA 6.

Both studies assessed patients’ HRQoL using the EQ-5D-3L and depression using the Patient Health Questionnaire 9 (PHQ-9). Also, medical professionals assessed the following clinical measures: ataxia severity using the Scale for Assessment and Rating of Ataxia (SARA) and the Inventory of Non-Ataxia Signs (INAS) at each follow-up visit [20, 21]. The activities of daily-living subscale of the Friedreich’s ataxia rating scale (FARS-ADL) and the Philadelphia Sleep Quality Index (PSQI) were further PROMs used in only one cohort study and, therefore, not employed in the final analysis. Similarly, only a subgroup of EUROSCA participants was assessed with the Montreal Cognitive Assessment (MoCA) for cognitive impairment and therefore not used in the analysis as well.

The measures used are described below.

### EuroQol European Quality of Life 5 Dimensions 3 Levels (EQ-5D-3L)

The EQ-5D-3L is one of the most used generic preference-based patient-reported outcome measures to assess patients' HRQoL [22]. A large body of literature confirms that the EQ-5D is a reliable and valid instrument for several neurodegenerative diseases [23]. The EQ-5D consists of five dimensions: mobility (MO), self-care (SC), usual activities (UA), pain/ discomfort (PD), and anxiety/ depression (AD). Each dimension is an item with three response levels: “1”, no problems; “2”, moderate problems; and “3”, extreme problems.

The descriptive system is used to create health states ranging from the best health state, “11111”, to the worst health state, “33333” [24]. With a value set of the responders’ country/region, health states are converted into a single utility index score. We tested the value sets for the two largest participant groups, England and Germany, and compared them to the European value set. The comparison showed that the English and German value sets showed significant differences in marginal effects especially in the anxiety/discomfort dimension which might influence the results. Based on these findings and to improve reliability, we decided to use the European value set instead to calculate the EQ-5D-3L utility indices [25]. The EQ-5D-3L also contains a visual analogue scale (EQ VAS) that asks participants to self-rate their health from 0 (worst imaginable health) to 100 (best imaginable health) [26].

### The Scale of the Assessment and Rating of Ataxia (SARA)

The Scale of the Assessment and Rating of Ataxia (SARA) is a validated and widely used clinical scale measuring ataxia severity [27]. SARA comprises eight items that focus on gait, stance, speech, finger-chase test, nose-finger test, fast alternating movements, and heel-shin test [21]. Clinical reviewers evaluate the items based on SCA patient’s performance. A sum score is created from item scores ranging from 0 (no ataxia) to 40 (most severe ataxia). Based on the SARA sum score, ataxia severity can be classified into “no to mild” (0–9.5), “moderate” (10–19.5), “strong” (20–29.5), and “extreme” (30–40) [21, 28].

### Inventory of Non-Ataxia Signs (INAS)

Apart from ataxia, numerous additional clinical signs are associated with SCA [17]. To capture these signs, the Inventory of Non-Ataxia Signs was used as a list of neurological symptoms that can evaluate the presence and severity of non-ataxia signs [27]. The INAS comprises 30 items that are grouped into 16 non-ataxia signs. The count of these signs is used to evaluate the severity, ranging from no to a maximum of 16 present signs. The INAS was validated and is widely used in various ataxia disorders [20].

### Patient Health Questionnaire (PHQ-9)

The Patient Health Questionnaire (PHQ-9) is part of the self-reported version of the PRIME-MD diagnostic instrument. It uses the 9-item depression module to diagnose depressiveness based on the Diagnostic and Statistical Manual of Mental Disorders IV (DSM IV) [29]. The scoring system has four levels ranging from “0” (not at all) to “3” (nearly every day). The nine items can be computed into a sum score for depression severity [30]. Depression severity ranges from “none to minimal” (0–4), “mild” (5–9), “moderate” (10–14), and “severe depression” (15–27) [29]. The PHQ-9 is widely validated and used for SCA and other various illnesses [11, 31].

### Statistical Analyses

#### Imputation Procedures

Only patients with complete baseline data were included. Missing follow-up values of the SARA, INAS, PHQ-9, and EQ-5D-3L utility index were imputed using multiple imputations via chained equation (MICE), adding 50 estimates for each missing, stratified for age, gender, SCA-type, and baseline values or values of the previous data assessment. Missing variables occurred for 3.1% of all variables but in 33.1% of all patients (see Supplementary Table 1) for 1-year, 2-year, and partly 3-year follow-up time points. The mean of all imputed estimates replaced each missing follow-up value [32]. This decision is based on Rösel et al. [33] which recommends multiple imputations for missing EQ-5D values when more than two time points are considered.

#### Bivariate Analyses

Based on the imputed patient sample, outcome measures (SARA, INAS, EQ-5D-3L utility index, and PHQ-9) were tested for correlation at baseline to evaluate the association of the continuous variables using Spearman rank correlations (r_s_). A correlation coefficient higher than 0.3 and 0.5 determines moderate or strong correlation, respectively [34]. Furthermore, means and standard deviation were calculated for each score at all time points and for score changes between baseline and follow-up. Paired *t*-tests and signed-rank tests were performed to assess the individual within-subject score changes over three years, and percentage changes based on mean change were also calculated.

#### Multivariable Analyses

Panel regression models were used to identify factors influencing the HRQoL over time. The panel regression models included baseline, 1-, 2-, and 3-year follow-up data. We used the EQ-5D-3L utility index as a dependent variable over time. Patients’ socio-demographics (age, gender, age of SCA onset), body mass index (BMI), SCA-type and SARA, INAS and PHQ-9, and RLS syndrome were used as independent variables.

As SCA 6 progressed clinically differently, we dichotomized the SCA type variable differentiating between SCA1, SCA2, SCA3, and SCA6. We tested for normal distribution of the EQ-5D-3L before the model index. The EQ-5D-3L index did not show skewed distributions or dominant floor or ceiling effects from baseline to 3-year follow-up.

#### Sensitivity Analyses

Linear regression models were performed as sensitivity analyses for the independent variables on EQ-5D-3L index and EQ VAS. Linear regression models examined the association between HRQoL and all listed independent variables from baseline to each of the three follow-up time points. Furthermore, linear regressions tested for the influence of study sites and SCA types on the patient sample to assess whether country or disease differences could affect the HRQoL progression.

All analyses were carried out using SAS (Version 9.4 M5, SAS Institute, Cary, NC, USA) and STATA (Version 16.0, Stata Corp, College Station, TX, USA).

## Results

### Sample

Table 1 shows patients’ characteristics. Fifty-three percent were female and, on average, 51 years old with a SARA score of 13 at baseline, indicating moderate ataxia severity. The mean SCA onset age was 40. SCA3 was most common with 34%, followed by SCA2 (29%), SCA6 (19%), and SCA1 (18%) (see supplementary table 2).

**Table 1:** Descriptive socio-demographic and clinical statistics at baseline (*n* = 344)

Socio-demographics	
*Women, n (%)*	183 (53.2)
*Age, mean (SD)*	50.45 (13.82)
18–29 years, *n* (%)	*23 (6.6)*
30–39 years, *n* (%)	*58 (16.9)*
40–49 years, *n* (%)	*92 (26.7)*
50–59 years, *n* (%)	*77 (22.4)*
60–69 years, *n* (%)	*56 (16.3)*
70–89 years, *n* (%)	*38 (11.1)*
*Body mass index (kg/m*^*2*^*), mean (SD)*	24.70 (4.03)
Underweight, *n* (%)	*14 (4.1)*
Normal weight, *n* (%)	*179 (49.7)*
Overweight, *n* (%)	*114 (33.1)*
Obese I, *n* (%)	*40 (11.6)*
Obese II/III, *n* (%)	*5 (1.5)*
*Age of onset*	40.38 (13.00)
*Less than 40 years*	*172 (50.0)*
*More than 40 years*	*172 (50.0)*
*Disease duration*	10.08 (5.84)
Quality of life	
*EQ-5D-3L index*	0.665 (0.185)
*EQ VAS*	64.45 (20.01)
Clinical measures	
*SARA sum score*	13.25 (6.83)
Mild ataxia severity (0*–*9), *n* (%)	*105 (30.5)*
Moderate ataxia severity (10*–*19), *n* (%)	*186 (54.1)*
Severe ataxia severity (20*–*29), *n* (%)	*41 (11.9)*
Extreme ataxia severity (30*–*40), *n* (%)	*12 (3.5)*
*INAS count*	3.90 (2.24)
Less than 4 symptoms, *n* (%)	*163 (47.4)*
More than 4 symptoms, *n* (%)	*181 (52.6)*
*PHQ9 sum score*	6.91 (6.93)
No depression (0*–*4)	*172 (50.0)*
Mild depression (5*–*9)	*102 (29.7)*
Moderate depression (10*–*14)	*33 (9.6)*
Severe depression (15*–*27)	*37 (10.8)*
*RLS symptoms*	17 (5.0)
SCA type	
*SCA1, n (%)*	63 (18.3)
*SCA2, n (%)*	100 (29.1)
*SCA3, n (%)*	116 (33.7)
*SCA6, n (%)*	65 (18.9)

^a^Only in the ESMI dataset; ^b^only in the EUROSCA dataset; *SCA* spinocerebellar ataxia, *SARA* Scale for the Assessment and Rating of Ataxia, *INAS* Inventory of Non-Ataxia Signs, *PHQ-9* Patient Health Questionnaire 9, *RLS* restless legs syndrome

### Correlation of HRQoL and Clinical Measures

**Fig. 2:**
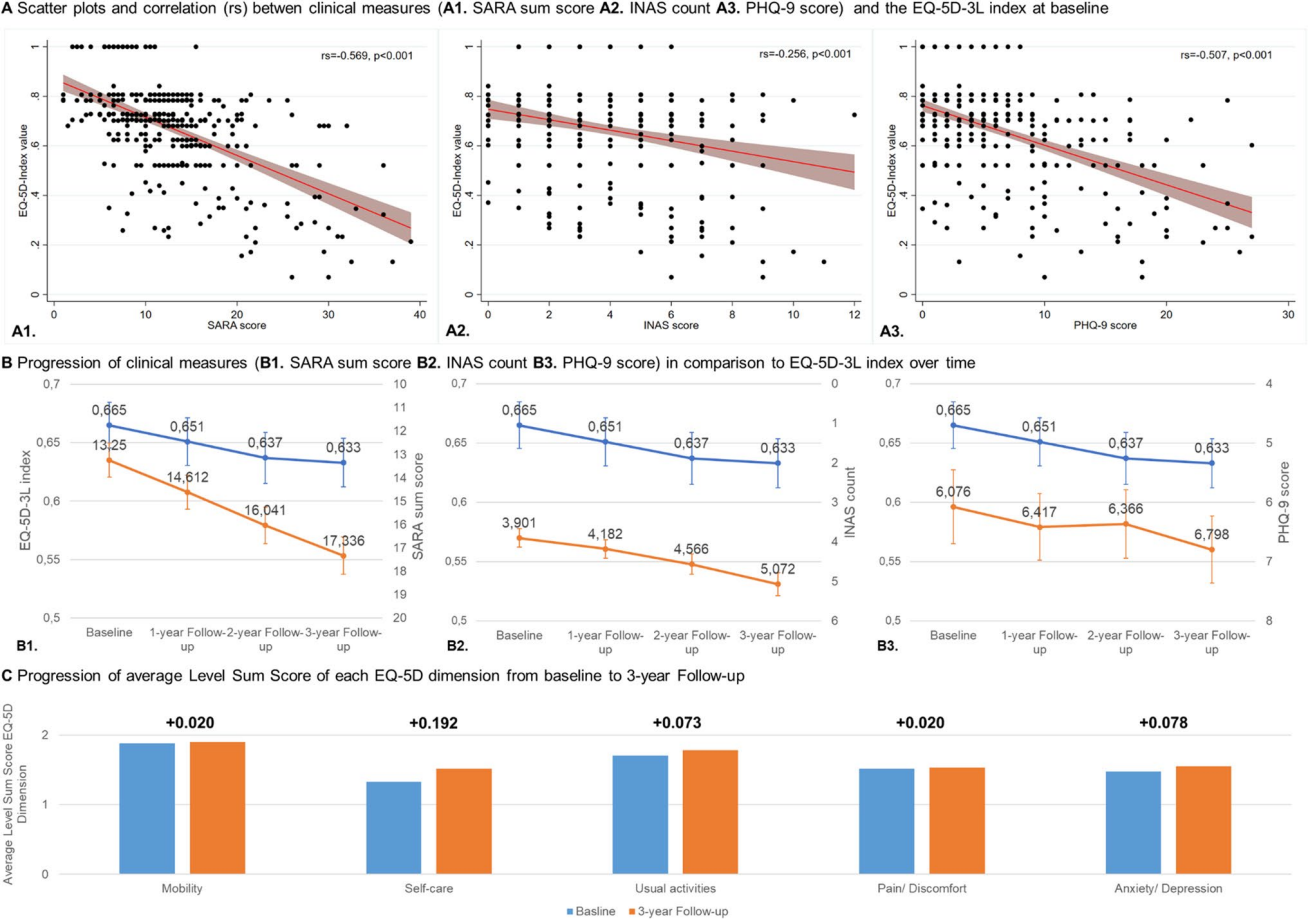
Overview of the correlation and progression of the EQ-5D-3L compared to clinical measures

HRQoL strongly correlated with ataxia severity (SARA: r_s_ = 0.569, *p* < 0.001) and depressive symptoms (PHQ-9: r_s_ = 0.507, *p* < 0.001) at baseline (see Fig. 2A).

Inverted means trends showed when comparing mean EQ-5D-3L index with clinical measures over time. The increase in patient depression over time (from 6.1 to 6.8, + 2.7%) was comparable to the reduction of the EQ-5D-3L utility index (− 3.2%). However, ataxia severity (SARA: from 13.3 to 17.3, + 10.2%) and non-ataxia signs (INAS: from 3.9 to 5.1, + 7.3%) increased more intensively compared to the percentage change of HRQoL. Fig. 2B and Table 2 demonstrate the difference in HRQoL compared to the clinical measures.

**Table 2:** Description of mean and mean difference of HRQoL and clinical measures at different annual time points (*n* = 344)

Variables	Time point	Mean	SD	Difference from specific time point to											
				1-Year follow-up				2-Year follow-up				3-Year follow-up			
				Mean	SD	%	*p Value*	Mean	SD	%	*p Value*	Mean	SD	%	*p* Value
EQ-5D-3L-index	Baseline	0.665	0.185												
	1-Year follow-up	0.651	0.193	− 0.014	0.160	− 2.11%	0.095┼	− 0.028	0.172	− 4.21%	0.003**	− 0.032	0.190	− 4.81%	0.002**
	2-Year follow-up	0.637	0.206					− 0.013	0.177	− 1.95%	0.159	− 0.018	0.184	− 2.71%	0.071┼
	3-Year follow-up	0.633	0.196									− 0.004	0.175	− 0.60%	0.6373
SARA sum score	Baseline	13.250	6.832												
	1-Year follow-up	14.612	6.924	1.362	2.754	10.28%	< 0.001***	2.791	3.121	21.06%	< 0.001***	4.086	3.846	30.84%	< 0.001***
	2-Year follow-up	16.041	7.374					1.429	2.288	10.78%	< 0.001***	2.725	3.005	20.57%	< 0.001***
	3-Year follow-up	17.336	7.634									1.296	2.537	9.78%	< 0.001***
INAS count	Baseline	3.901	2.242												
	1-Year follow-up	4.182	2.248	0.280	1.644	7.18%	0.002**	0.665	1.933	17.05%	< 0.001***	1.171	2.13	30.02%	< 0.001***
	2-Year follow-up	4.566	2.475					0.384	1.509	9.84%	< 0.001***	0.891	1.799	22.84%	< 0.001***
	3-Year follow-up	5.072	2.714									0.506	1.56	12.97%	< 0.001***
PHQ-9 sum score	Baseline	6.076	5.873												
	1-Year follow-up	6.417	5.323	0.341	4.426	5.61%	0.154	0.291	4.770	4.79%	0.259	0.722	4.891	11.88%	0.006**
	2-Year follow-up	6.366	5.459					− 0.05	4.24	-0.82%	0.826	0.381	4.373	6.27%	0.107
	3-Year follow-up	6.798	5.381									0.431	3.922	7.09%	0.042*
EQ VAS score*	Baseline	64.452	20.011												
	1-Year follow-up	63.064	22.431	− 1.529	22.468	− 2.37%	0.2173	− 6.17	20.325	− 9.57%	< 0.001***	− 5.575	20.668	− 8.65%	< 0.001***
	2-Year follow-up	58.15	22.553					− 4.564	21.128	− 7.08%	< 0.001***	− 3.950	20.711	− 6.13%	< 0.001***
	3-Year follow-up	58.728	20.825									0.262	17.343	0.41%	0.783

*n = 336; score ranges: EQ-5D-3L-Index (0.035*–*1.000), SARA sum score (0.000*–*40.000), INAS count (0.000–12.000); PHQ-9 sum score (0.000–27.000), EQ VAS score (0.000*–*100.000); higher values indicate higher HRQoL (EQ-5D-3L-Index), higher ataxia severity (SARA sum score), higher sum of non-ataxia symptoms (INAS count), higher depression (PHQ-9 sum score), higher self-rated health (EQ-VAS score); ┼, *,**, and *** = levels of significance (0.10–0.05, < 0.05, <0.01, <0.001)

### Description of HRQoL and Clinical Measures Over Time

HRQoL (EQ-5D-3L utility index) decreased significantly from 0.665 to 0.633 (− 0.032 (− 3.2%), SD 0.190, *p* = 0.002) between baseline and the 3-year follow-up, with an average annual decrease of 0.011. The change in HRQoL over time is shown in Fig. 2B and Table 2. When comparing EQ-5D-3L dimensions at baseline and after three years, the average level score of the dimension mobility and pain/discomfort demonstrated high disability at baseline but did not appear to change significantly (+ 0.02, respectively). Usual activities also showed high disability at baseline. Contrary to this, the other domains showed low disabilities at baseline and a decline over time. Anxiety/depression and usual activities declined moderately (+ 0.078 and + 0.073, respectively), and self-care deteriorated severely (+ 0.192) over 3 years (see Fig. 2C). Looking at clinical measures, SARA, INAS, and PHQ-9 significantly increased between baseline and the 3-year follow-up.

Looking at different patient subgroups, HRQoL more intensively decreased over time in male, younger, more depressed, under- /over-weight patients and patients with earlier disease onset and mild ataxia severity. The most intensive decline of HRQoL was seen for patients aged 18–29 (− 14.3%), with a BMI between 30 and 35 (− 11.4%), a disease onset before age 40 (− 9.4%) and with mild ataxia severity at baseline (− 6.9%) (see Supplementary Table 2).

### Factors Influencing HRQoL Progression

Multivariable panel regression models confirmed subgroup analyses. Higher BMI (β = − 0.0031; CI^95^= [− 0.0060; − 0.0003]; *p* = 0.029), male gender (β = − 0.0240; CI^95^ [− 0.0460; − 0.0013]; *p* = 0.038), lower age of onset (β = 0.0022; CI^95^ = [− 0.0001; 0.0045]; *p* = 0.058), and higher increase in SARA (β = − 0.0096; CI^95^ = [− 0.0113; − 0.0080]; *p* ≤ 0.001), and PHQ-9 score (β =− 0.0120; CI^95^ = [− 0.0142; − 0.0107]; *p* ≤ 0.001) significantly reduced the EQ-5D-3L index over time. The panel regression model had very high predictability in-between time points (*r*^2^ = 0.598) and overall (*r*^2^ = 0.429) (see Table 3).

**Table 3:** Panel random effects (variable time) regression model analysing factors influencing HRQOL (EQ-5D-3L index) over time from baseline to 3-year follow-up (*n* = 344)

Variables	ß	SE	CI^95^	*p* Value
Age	− *0.0017*	*0.0010*	[− 0.0037; 0.0003]	*0.103*
BMI	− 0.0031	0.0014	[− 0.0060; − 0.0003]	0.029*
Male gender (reference female)	− 0.0240	0.0114	[− 0.0460; − 0.0013]	0.038*
Age of onset	0.0022	0.0012	[− 0.0001; 0.0045]	0.058^┼^
Change of SARA between visits	− 0.0096	0.0008	[− 0.0113; − 0.008]	< 0.001***
Change of INAS between visits	− 0.0023	0.0024	[− 0.0070; 0.0023]	0.329
Change of PHQ-9 between visits	− 0.0120	0.0009	[− 0.0142; − 0.0107]	< 0.001***
SCA type 6 (ref. SCA 1, 2, 3)	− 0.0230	0.0186	[− 0.0595; 0.0133]	0.214
RLS syndrome	− 0.0200	0.0265	[− 0.0720; 0.0319]	0.448
Intercept	0.9981	0.0456	[0.9087; 1.0876]	< 0.0001
*R*^2^				
*Within*	0.078			
*Between*	0.598			
*Overall*	0.429			

Max observations per group 4, Overall model significance *p* < 0.0001; ^**┼**^, *,**, and *** = levels of significance (0.10–0.05, < 0.05, < 0.01, < 0.001)

Linear regressions evaluating the impact of the independent variables on EQ-5D-3L index and EQ VAS from baseline to 1-, 2- and 3-year follow-up confirmed findings of the panel regression analyses and suggested that age and RLS might have an impact on HRQoL progression at specific time points (see Supplementary Table 3 and 5).

## Discussion

### Summary of Results

The study demonstrated for the first time HRQoL progression compared to disease-specific and generic health-related measures in SCA and identified modifiable and non-modifiable factors in longitudinal data from two international cohorts. SCA patients’ HRQoL decreased steadily over three years. This decline significantly correlated with clinical outcomes like ataxia severity and non-ataxia signs. The panel regression results showed a negative impact of higher depression and BMI on HRQoL. Lower ataxia severity, male gender, and an early onset of SCA disorders negatively affected patients’ HRQoL over time. Our findings add evidence about factors influencing HRQoL in SCA patients by revealing potentially modifiable factors, like BMI and depression, as predictors of a more intensive HRQoL decline over time. Furthermore, the association between HRQoL progression and ataxia severity showed that SARA is especially meaningful and relevant for patients predicting the change in HRQoL over the disease course.

### Modifiable Predictors of HRQoL

Patients’ depression can significantly modify overall HRQoL progression. Major depression is frequent in SCA [11]. The data showed that depression treatment could possibly inhibit HRQoL progression; however, depression affecting HRQoL in SCA has not yet been thoroughly investigated. Min-Ting Lin et al. [35] suggested depression as a part of neurodegeneration in SCA. Silva et al. advocated for more attention and interventions regarding depression [36]. Our results extended these findings, indicating the impact of depression on patients’ future HRQoL [37, 38] Unlike common findings, we could not find differences between genders and depression. Furthermore, Hoche et al. [39] discussed possible associations between cognitive impairment and depressive symptoms as part of the Cerebellar Cognitive Affective/Schmahmann syndrome scale (CCAS). Cognitive Impairment can be associated with depression. However, due to the limited cognitive assessment in both cohort studies with the MoCA only being used in a subset of patients, our analysis could not evaluate the influence of cognitive impairment on HRQoL and maybe confounding the impact of depression. Future cohort studies should include the CCAS to better understand the association between cognitive impairment, depressive symptoms, and HRQoL. Additionally, Hsu et al. [40] underlined that depression is associated with insomnia and that improving both depression and insomnia could improve SCA patients’ HRQoL. Further research is needed to investigate if and to what extent concomitant treatment of depression and sleep disturbance in SCA could affect HRQoL in SCA patients.

Our data shows that BMI could be another modifiable HRQoL determinant in both directions: underweight and obesity. Our findings revealed that a higher BMI is associated with a stronger HRQoL decline. However, baseline HRQoL of underweight SCA patients is already significantly lower than the average HRQoL of SCA patients. When left untreated, weight influence could negatively affect SCA patients’ HRQOL progression. The adverse effects of both obesity and underweight as disease progression modifiers have been discussed in SCA2 and SCA3 patients [41, 42]. Our findings show that underweight and obesity are possible predictors for HRQoL progression in patients with SCA1, SCA2, SCA3, and SCA6. Diallo et al. showed that BMI decline is related to SCA progression, raising the question that obese SCA patient subgroups might not profit from weight management [41, 42]. Further research is needed to evaluate the impact of BMI management on HRQoL in SCA patients.

Additionally, SCA patients might adapt to some ataxia symptoms over time but struggle increasingly with other not-ataxia-related symptoms like depression and limitations in their everyday activities in more severe disease stages. This is seen in the significant change in the EQ-5D-3L’s self-care and depression/anxiety dimensions, implying an increasing lack of independence. These findings contribute to Lo et al. [48], stating that depression is not simply caused by SCA diseases. The association of the self-care dimension and PHQ-9 implies that a growing lack of independence and loss of self-care might cause negative HRQoL progression. Similar findings can be found in other neurodegenerative diseases, like late-stage Parkinson’s disease [47] and a small 15 SCA10 patient study. Reis Santos et al. [49] showed an association between functional dependence in activities of daily living and lower HRQoL. This raises the question of how the relationship between caregiver and SCA patient changes over the course of the disease.

### Non-modifiable Confounder of HRQoL

Gender plays a significant role in HRQoL progression for SCA patients. Male SCA patients showed a significantly lower HRQoL and a more substantial HRQoL decline. Similar findings were observed in Parkinsons' disease. Several authors reported on the impact of gender on HRQoL and life expectancy in Parkinson’s disease [43, 44]. While men suffer primarily from the progression of motor symptoms, women are more frequently affected by depression and anxiety, impacting HRQoL. Our study is the first to show gender differences in HRQoL for SCA. Female patients’ HRQoL is less affected by disease progression. Similarly, female patients struggle less with ataxia-specific symptoms and more with non-ataxia symptoms as shown by several studies [45–47]. These findings could imply that clinical therapies need to be gender-specific.

Diallo et al. (2018) [4] and Jacobi et al. (2015) [5] showed that SCA1, SCA2, and SCA3 patients have a faster disease progression than SCA 6 patients. Our results similarly show that HRQoL appears to have a less intensive decrease over time in SCA6 compared to SCA1–SCA3 patients. However, subgroup analysis showed that despite slower progression, factors influencing HRQoL progression appear similar in all four SCA disorders.

An association between age and change in HRQoL between baseline and 1-year and 2-year follow-up time points was found. However, age was not significant in our panel regression. Perez-Flores et al. [50] showed that age was a significant predictor of HRQOL in patients with Friedreich Ataxia. Our analysis confirmed this, demonstrating that older age might be associated with a more intensive decline in patients' HRQoL [50].

### Strengths and Limitations

SCA disorders are rare, and this generally leads to studies with small patient samples. Studies evaluating HRQoL in SCA are lacking. By merging two of the largest recent cohort studies, it was possible to create a longitudinal study sample large enough to identify factors influencing HRQoL in rare SCA. Additionally, a European value set was used compared to several national value sets, possibly restricting the preciseness of the study sample.

We found differences in diagnosis and measurement of RLS in both samples, leading to an exclusion of RLS in the main analysis.

Merging data sets from different cohorts leads to several limitations. In our study, it led to only the main clinical measures and some sociodemographic factors to be taken into account, which might limit the range of results. The restriction to use baseline BMI shows this limitation. Diallo et al. showed that BMI decline significantly impacts ataxia severity, possibly leading to an overestimation of obesity in our analysis [51]. Further studies should include further clinical measures and lifestyle factors to identify additional modifiable factors for future person-centred treatment plans.

We decided to include eight patients in our study sample with premanifest stages of cerebellar syndrome because of pre-existing clinical recruitment in both studies. Furthermore, we performed tests to identify the impact of these patients on the analysis. While these patients did not impact the results, future studies might be advised to clearly differentiate between pre-manifest and manifest stages of disease.

Additionally, we were looking at several disorders at a time (supplementary table 4). While SCA1, SCA2, and SCA3 appear comparable, SCA6 significantly influences the HRQoL progression, which might lead to overestimating or underestimating the effect of other influencing factors. Another limitation is the time difference between both data sets. Over 15 years, changes in symptomatic treatment or health services might have affected the course of the disease and HRQoL progression, limiting the generalizability of the presented results. We also could not track the impact of disease duration in our study. We also could not track the impact of cognitive impairment frequent in cerebellar ataxias due to limited cognitive assessment in both cohort studies which might lead to over or underestimation of some of the factors found.

As population weights were not employed, age-related HRQoL decline might have also overestimated our findings. Furthermore, newer versions of questionnaires, such as the EQ-5D-5L with five levels and further disease-specific HRQoL, and patient-reported outcome measures, such as the PROM-Ataxia [52], have been employed and might better capture HRQoL in SCA patients. Whereas the EQ-5D-3L index outperforms the EQ-VAS, Janssen et al. showed that the EQ VAS underperforms in responsiveness and the EQ-5D-3L index can overestimate health problems compared to the EQ-5D-5L which should be the preferred HRQoL measure in future studies.

Both cohort studies were primarily performed in European countries. Thus, differences between countries and continents might limit generalizability with a significant amount of SCA patients being located in South America [53].

## Summary

This is one of the first studies demonstrating the progression of HRQoL and identifying the main predicting factors of HRQoL over time in SCA. Our findings revealed that a higher depression score, BMI, and ataxia severity significantly negatively affect SCA patients’ HRQoL. Male patients and patients with an earlier SCA disease onset demonstrated a more pronounced decline of HRQoL. Overall, the plurality of unmodifiable and modifiable factors shows a significant overall impact on the everyday life of SCA patients and suggests that many patients not only clinically suffer but also struggle psychosocially with the ongoing disease [54]. A stronger focus on modifiable and potentially treatable factors, like mental health, obesity, and underweight, could help clinical evaluation and treatment of SCA diseases and improve health and well-being in patients with SCA. Further research is needed to implement and evaluate multicomponent interventions that address these modifiable factors in SCA to improve patients’ HRQoL and everyday life. Future studies are also needed to investigate how imaging or genetic evaluation such as CAG repeat expansions would allow for predictions on the progression of HRQoL.

### Supplementary information

ESM 1 (DOCX 91.7 kb)

## Data Availability

No datasets were generated or analysed during the current study.

## Abbreviations

CCAS: Cerebellar cognitive affective/Schmahmann syndrome scale
ESMI: European Spinocerebellar Ataxia Type 3/Machado-Joseph Disease Initiative
EUROSCA: European Spinocerebellar Ataxia Natural History Study
SARA: Scale for the Assessment and Rating of Ataxia
INAS: Inventory of Non-Ataxia Signs
PHQ-9: Patient Health Questionnaire 9
DSM-IV: Diagnostic and Statistical Manual of Mental Disorders, Fourth Edition
EQ VAS: (EuroQol) European Quality of Life Visual Analogue Scale
EQ-5D-3L: (EuroQol) European Quality of Life 5 Dimensions 3 Levels
FARS-ADL: Friedreich’s Ataxia Rating Scale – Activities of Daily Living
HRQoL: Health-related Quality-of-Life
MoCA: Montreal cognitive assessment
PRO: Patient-reported outcome
PROM: Patient-reported outcome measurement
PSQI: Philadelphia Sleep Quality Index
RLS: Restless legs syndrome
SCA: Spinocerebellar ataxia
UHDRS: Unified Huntington Disease Rating Scale
