# Supplementary material for: Factors Influencing Health-Related Quality of Life of Patients with Spinocerebellar Ataxia
Source: Cerebellum. 2024 Jan 27;23(4):1466–77. doi: 10.1007/s12311-024-01657-2 (PMC11269494; doi:10.1007/s12311-024-01657-2)
Supplement: Supplementary file 1 — (DOCX 91.7 kb) [file 12311_2024_1657_MOESM1_ESM.docx]

**SUPPLEMENTARY**

**Supplementary Table 1.** Overview of existing variables from Baseline to 3-year follow-up. Percentage indicates the amount of all existing variables based on the patient sample. Last selection (n=344) was used for the imputation and panel regression.

| **Baseline** | | | | **1-year follow-up** | | | | **2-year follow-up** | | | | **3-year follow-up** | | | | **Other variables** | | |
| --- | --- | --- | --- | --- | --- | --- | --- | --- | --- | --- | --- | --- | --- | --- | --- | --- | --- | --- |
| **EQ-5D index** | **SARA score** | **INAS count** | **PHQ-9 score** | **EQ-5D index** | **SARA score** | **INAS count** | **PHQ-9 score** | **EQ-5D index** | **SARA score** | **INAS count** | **PHQ-9 score** | **EQ-5D index** | **SARA score** | **INAS count** | **PHQ-9 score** | **Age, Sex, BMI, Age of Onset, RLS** | | |
|  |  |  |  |  |  |  |  |  |  |  |  |  |  |  |  |  | | |
| **835** | 838 | 754 | 787 | **703** | 696 | 586 | 645 | **571** | 567 | 525 | 532 | **435** | 434 | 403 | 411 | 763 | | |
|  |  |  |  |  |  |  |  |  |  |  |  |  |  |  |  |  | | |
| x | x | x | x |  |  |  |  |  |  |  |  |  |  |  |  |  |  | |
| **598** | 71.6% |  |  |  |  |  |  |  |  |  |  |  |  |  |  |  |  | |
| x | x | x | x | x | x | x | x |  |  |  |  |  |  |  |  |  |  | |
|  |  |  |  | **389** | 55.5% |  |  |  |  |  |  |  |  |  |  |  |  | |
| x | x | x | x | x | x | x | x | x | x | x | x |  |  |  |  |  |  | |
|  |  |  |  |  |  |  |  | **351** | 61.5% |  |  |  |  |  |  |  |  | |
| x | x | x | x | x | x | x | x | x | x | x | x | x | x | x | x |  |  | |
|  |  |  |  |  |  |  |  |  |  |  |  | **236** | 54.2% |  |  |  |  | |
| x | x | x | x | x | x | x | x | x | x | x | x | x | x | x | x | x | | |
|  |  |  |  |  |  |  |  |  |  |  |  | **230** | 52.9% |  |  |  | |  |
| x | x | x | x |  |  |  |  |  |  |  |  | x | x | x | x | x | | |
|  |  |  |  |  |  |  |  |  |  |  |  | **317** | 72.9% |  |  |  | |  |
| x | x | x | x |  |  |  |  |  |  |  |  | x |  |  |  |  |  | |
|  |  |  |  |  |  |  |  |  |  |  |  | **356** | 81.8% |  |  |  |  | |
| x | x | x | x |  |  |  |  |  |  |  |  | x |  |  |  | x | | |
|  |  |  |  |  |  |  |  |  |  |  |  | **344** | 79.1% |  |  |  | |  |

|  |  |  | **Baseline** | | **3-year Follow-up** | | **Difference baseline - 3-year follow-up** | | | |
| --- | --- | --- | --- | --- | --- | --- | --- | --- | --- | --- |
| **Variable** | **Subgroup** | **n** | mean | SD | mean | SD | mean | SD | % | *p-value* |
| **Gender** | Female | 183 | 0.690 | 0.179 | 0.662 | 0.189 | -0.028 | 0.180 | -4.06% | **0.036** |
|  | Male | 161 | 0.637 | 0.189 | 0.600 | 0.200 | -0.037 | 0.200 | -5.81% | **0.020** |
| **Age** | 18 - 29 years | 23 | 0.673 | 0.193 | 0.578 | 0.215 | -0.096 | 0.154 | -14.26% | **0.007** |
|  | 30 - 39 years | 58 | 0.690 | 0.153 | 0.621 | 0.218 | -0.069 | 0.227 | -10.00% | **0.025** |
|  | 40 - 49 years | 92 | 0.670 | 0.182 | 0.622 | 0.189 | -0.048 | 0.169 | -7.16% | **0.008** |
|  | 50 - 59 years | 77 | 0.660 | 0.183 | 0.655 | 0.178 | -0.005 | 0.178 | -0.76% | 0.793 |
|  | 60 - 69 years | 56 | 0.660 | 0.221 | 0.671 | 0.197 | 0.011 | 3.121 | 1.67% | 0.674 |
|  | 70 - 89 years | 38 | 0.630 | 0.188 | 0.609 | 0.200 | -0.020 | 0.193 | -3.17% | 0.523 |
| **Body Mass Index** | Underweight | 14 | 0.552 | 0.234 | 0.517 | 0.259 | -0.035 | 0.148 | -6.34% | 0.395 |
|  | Normal weight | 171 | 0.689 | 0.170 | 0.641 | 0.197 | -0.049 | 0.191 | -7.11% | **0.001** |
|  | Overweight | 114 | 0.668 | 0.186 | 0.649 | 0.187 | -0.020 | 0.173 | -2.99% | 0.222 |
|  | Obese I | 40 | 0.593 | 0.209 | 0.601 | 0.194 | 0.008 | 0.241 | 1.35% | 0.826 |
|  | Obese II | 5 | 0.683 | 0.103 | 0.605 | 0.128 | -0.078 | 0.087 | -11.42% | 0.116 |
| **Age of Onset** | Below 40 years | 172 | 0.661 | 0.182 | 0.599 | 0.209 | -0.062 | 0.193 | -9.38% | **<0.001** |
|  | Above 40 years | 172 | 0.670 | 0.189 | 0.677 | 0.176 | -0.003 | 0.183 | -0.45% | 0.837 |
| **SCA type** | SCA 1, 2, 3 | 279 | 0.669 | 0.189 | 0.632 | 0.201 | -0.037 | 0.191 | -5.53% | **0.001** |
|  | SCA 6 | 65 | 0.650 | 0.170 | 0.637 | 0.176 | -0.014 | 0.185 | -2.15% | 0.544 |
| **SARA Sum Score** | Mild ataxia severity | 105 | 0.756 | 0.147 | 0.705 | 0.164 | -0.052 | 0.150 | -6.88% | **0.006** |
|  | Moderate ataxia severity | 186 | 0.673 | 0.142 | 0.636 | 0.171 | -0.037 | 0.283 | -5.50% | **0.006** |
|  | Severe ataxia severity | 41 | 0.496 | 0.215 | 0.536 | 0.243 | 0.040 | 0.280 | 7.86% | 0.370 |
|  | Extreme ataxia severity | 12 | 0.320 | 0.205 | 0.288 | 0.156 | -0.032 | 0.204 | -10.00% | 0.601 |
| **INAS Count** | Less than 4 symptoms | 163 | 0.686 | 0.172 | 0.664 | 0.188 | -0.022 | 0.179 | -3.21% | 0.115 |
|  | More than 4 symptoms | 181 | 0.647 | 0.195 | 0.605 | 0.200 | -0.041 | 0.199 | -6.34% | **0.006** |
| **PHQ-9 Sum Score** | No depression | 172 | 0.731 | 0.147 | 0.694 | 0.184 | -0.037 | 0.167 | -5.1% | **0.004** |
|  | Mild depression | 102 | 0.670 | 0.158 | 0.610 | 0.163 | -0.060 | 0.175 | -8.90.5% | **<0.001** |
|  | Moderate depression | 33 | 0.567 | 0.181 | 0.566 | 0.172 | 0.001 | 0.202 | -0.17% | 0.984 |
|  | Severe depression | 37 | 0.435 | 0.200 | 0.474 | 0.236 | 0.039 | 0.282 | 9.0% | 0.406 |

**Supplementary Table 2.** Mean HRQOL (EQ-5D-3L utility index) at baseline and 3-year follow-up and the difference for patient subgroups (age, gender, SCA Type, age of onset, ataxia severity - SARA Sum Score, non-ataxia signs - INAS Count, depression severity - PHQ-9 Sum Score)

**Supplementary Table 3.** Multivariate linear regression model analysing factors influencing EQ-5D-3L index from baseline to each follow-up using differences (deltas) (n=344)

|  | **Baseline to 1-year Follow-up^1^** | | **Baseline to 2-year Follow-up^2^** | | **Baseline to 3-year Follow-up^3^** | |
| --- | --- | --- | --- | --- | --- | --- |
|  | ß (SE) | CI^95^ | ß (SE) | CI^95^ | ß (SE) | CI^95^ |
| Age | **-0.004** (0.001)** | [-0.007;-0.002] | **-0.005** (0.001)** | [-0.008;-0.002] | -0.002 (0.002) | [-0.005;0.002] |
| BMI | -0.002 (0.002) | [-0.006;0.001] | 0.000 (0.002) | [-0.004;0.004] | 0.001 (0.002) | [-0.004;0.005] |
| Female gender (reference male) | **0.006*** (0.001)** | [0.003;0.009] | **0.005** (0.002)** | [0.002;0.009] | **0.004* (0.002)** | [0;0.007] |
| Age of Onset | 0.001(0.015) | [-0.029;0.032] | -0.008 (0.017) | [-0.04;0.025] | **-0.037* (0.018)** | [-0.072;-0.002] |
| Change of SARA between visits | **-0.009** (0.003)** | [-0.014;-0.003] | **-0.012*** (0.003)** | [-0.018;-0.006] | -0.001 (0.003) | [-0.006;0.004] |
| Change of INAS between visits | 0.008 (0.005) | [-0.002;0.017] | 0.000 (0.004) | [-0.009;0.008] | -0.007 (0.005) | [-0.016;0.002] |
| Change of PHQ-9 between visits | **-0.007*** (0.002)** | [-0.01;-0.003] | **-0.007*** (0.002)** | [-0.011;-0.004] | **-0.008*** (0.002)** | [-0.012;-0.004] |
| SCA type 6  (ref. SCA 1, 2, 3) | 0.045 (0.024) | [-0.002;0.092] | 0.051 (0.027) | [-0.002;0.092] | 0.045 (0.021) | [-0.002;0.092] |
| RLS syndrome | **-0.079**** (0.036) | [-0.149;-0.009] | -0.050 (0.041) | [-0.131;0.031] | **-0.079* (0.036)** | [-0.149;-0.009] |

*,**, *** - levels of significance (<0.05, <0.01, <0.001); ^1^ R^2^ 0.258; Adjusted R^2^ 0.236; p = 0.03 ; ^2^ R^2^ 0.244; Adjusted R^2^ 0.22; p = 0.04 ; ^3^ R^2^ 0.294; Adjusted R^2^ 0.273; p = <0.0001

| **Supplementary Table 4.** Descriptive Socio-demographic and Clinical Statistics at Baseline (n = 344) | | | | | | |
| --- | --- | --- | --- | --- | --- | --- |
| **Sociodemographics** | **All patients** | **SCA1** (n = 63) | **SCA2** (n=100) | **SCA3** (n=116) | **SCA6** (n=65) |  |
| Women, n (%) | 183 (53.2) | 43 (68.3) | 54 (54.0) | 54 (46.6) | 33 (50.77) |  |
| Age, mean (SD) | 50.45 (13.82) | 43.79 (11.98) | 45.10 (12.79) | 50.4 (11.2) | 65.15 (10.0) |  |
| *18-29 years, n (%)* | *23 (6.6)* | *8 (12.7)* | *12 (12.0)* | *3 (2.6)* | *0 (0.0)* |  |
| *30-39 years, n (%)* | *58 (16.9)* | *16 (25.4)* | *21 (21.0)* | *19 (16.4)* | *2 (3.1)* |  |
| *40-49 years, n (%)* | *92 (26.7)* | *20 (31.8)* | *32 (32.0)* | *35 (30.2)* | *5 (7.7)* |  |
| *50-59 years, n (%)* | *77 (22.4)* | *12 (19.1)* | *4 (4.0)* | *30 (25.9)* | *9 (13.9)* |  |
| *60-69 years, n (%)* | *56 (16.3)* | *6 (9.5)* | *4 (4.0)* | *23 (19.8)* | *23 (35.4)* |  |
| *70-89 years, n (%)* | *38 (11.1)* | *1 (1.6)* | *1 (1.0)* | *6 (5.2)* | *26 (40.0)* |  |
| Body mass index (kg/m^2^), mean (SD) | 24.7 (4.03) | 25.1 (4.01) | 25.30 (11.90) | 23.41 (3.6) | 25.89 (4.06) |  |
| *Underweight, n (%)* | *14 (4.1)* | *2 (3.2)* | *2 (2.0)* | *10 (8.6)* | *0 (0.0)* |  |
| *Normal weight, n (%)* | *179 (49.7)* | *28 (44.4)* | *46 (46.0)* | *70 (60.3)* | *27 (41.5)* |  |
| *Overweight, n (%)* | *114 (33.1)* | *20 (31.8)* | *38 (38.0)* | *28 (24.1)* | *28 (43.1)* |  |
| *Obese I, n (%)* | *40 (11.6)* | *12 (19.1)* | *11 (11.0)* | *8 (6.9)* | *9 (13.95)* |  |
| *Obese II/III, n (%)* | *5 (1.5)* | *1 (1.6)* | *3 (3.0)* | *0 (0.0)* | *1 (1.54)* |  |
| Age of onset | 40.38 (13.0) | 36.06 (10.97) | 36.06 (10.97) | 39.57 (9.53) | 55.34 (9.98) |  |
| *Less than 40 years* | *172 (50.0)* | *24 (38.1)* | *70 (70.0)* | *57 (49.1)* | *6 (9.2)* |  |
| *More than 40 years* | *172 (50.0)* | *39 (61.9)* | *30 (30.0)* | *59 (50.9)* | *59 (90.8)* |  |
| Disease Duration | *10.08 (5.84)* | *7.73 (4.47)* | *10.79 (5.83)* | *10.88 (6.32)* | *9.82 (5.63)* |  |
| **Quality of Life** |  |  |  |  |  |  |
| EQ-5D-3L index | 0.665 (0.185) | 0.662 (0.159) | 0.653 (0.197) | 0.686 (0.197) | 0.650 (0.170) |  |
| EQ VAS | 64.45 (20.01) | 64.73 (19.81) | 65.36 (19.77) | 64.22 (20.96) | 63.19 (19.21) |  |
| **Clinical measures** |  |  |  |  |  |  |
| SARA sum score | 13.25 (6.83) | 12.31 (6.18) | 12.31 (6.18) | 11.79 (6.66) | 14.47 (5.96) |  |
| *Mild ataxia severity (0-9), n (%)* | *105 (30.5)* | *21 (33.3)* | *29 (29.0)* | *44 (37.9)* | *11 (16.9)* |  |
| *Moderate ataxia severity (10-19), n (%)* | *186 (54.1)* | *35 (55.6)* | *52 (52.0)* | *55 (47.4)* | *44 (67.7)* |  |
| *Severe ataxia severity (20-29), n (%)* | *41 (11.9)* | *8 (9.5)* | *13 (13.0)* | *15 (12.9)* | *7 (10.8)* |  |
| *Extreme ataxia severity (30-40), n (%)* | *12 (3.5)* | *1 (1.6)* | *6 (6.0)* | *2 (1.7)* | *3 (4.6)* |  |
| INAS count | 3.90 (2.24) | 4.46 (2.05) | 4.08 (2.07) | 4.47 (2.24) | 2.06 (1.67) |  |
| *Less than 4 symptoms, n (%)* | *163 (47.4)* | *22 (34.92)* | *46 (46.0)* | *40 (34.48)* | *55 (84.62)* |  |
| *More than 4 symptoms, n (%)* | *181 (52.6)* | *41 (65.1)* | *54 (54.0)* | *76 (65.52)* | *10 (15.38)* |  |
| PHQ9 sum score | 6.91 (6.93) | 6.71 (6.72) | 5.08 (4.89) | 6.80 (6.06) | 5.69 (5.92) |  |
| *No depression (0-4)* | *172 (50.0)* | *32 (50.8)* | 55 (55.0) | *48 (41.4)* | *37 (56.92)* |  |
| *Mild depression (5-9)* | *102 (29.7)* | *17 (27.0)* | *29 (29.0)* | *39 (33.6)* | *17 (26.15)* |  |
| *Moderate depression (10-14)* | *33 (9.6)* | *5 (7.9)* | *8 (8.0)* | *16 (13.8)* | *4 (6.15)* |  |
| *Severe depression (15-27)* | *37 (10.8)* | *9 (14.3)* | *8 (8.0)* | *13 (11.2)* | *7 (10.77)* |  |
| RLS symptoms | 17 (5.0) | 2 (3.1) | 3 (3.0) | 9 (7.7) | 3 (4.6) |  |
| **SCA-Type** |  |  |  |  |  |  |
| SCA1, n (%) | 63 (18.3) | 63 (100.0) |  |  |  |  |
| SCA2, n (%) | 100 (29.1) |  | 100 (100.0) |  |  |  |
| SCA3, n (%) | 116 (33.7) |  |  | 116 (100) |  |  |
| SCA6, n (%) | 65 (18.9) |  |  |  | 65 (100) |  |

**Supplementary Table 5.** Mean EQ VAS at baseline and 3-year follow-up and the difference for patient subgroups (age, gender, SCA Type, age of onset, ataxia severity - SARA Sum Score, non-ataxia signs - INAS Count, depression severity - PHQ-9 Sum Score), number varies from baseline (n=344) to 3-year follow-up (n=335)

|  |  |  | **Baseline** | | **3-year Follow-up** | | **Difference baseline - 3-year follow-up** | | | |
| --- | --- | --- | --- | --- | --- | --- | --- | --- | --- | --- |
| **Variable** | **Subgroup** | **n** | mean | SD | mean | SD | mean | SD | % | *p-value* |
| **Gender** | Female | 183 (179) | 64.29 | 20.38 | 57.93 | 20.75 | -6.51 | 20.70 | -10.13% | **<0.001** |
|  | Male | 161 (156) | 64.64 | 19.64 | 59.65 | 20.94 | -4.50 | 20.65 | -6.96% | **0.007** |
| **Age** | 18 - 29 years | 23 (23) | 60.61 | 22.81 | 56.61 | 21.22 | -4.00 | 25.42 | -6.60% | 0.458 |
|  | 30 - 39 years | 58 (56) | 66.47 | 20.52 | 60.00 | 20.10 | -5.45 | 18.35 | -8.19% | **0.003** |
|  | 40 - 49 years | 92 (91) | 64.54 | 19.82 | 58.30 | 20.91 | -6.19 | 21.16 | -9.59% | **0.006** |
|  | 50 - 59 years | 77 (73) | 64.04 | 19.52 | 60.34 | 19.79 | -3.51 | 18.28 | -5.48% | 0.106 |
|  | 60 - 69 years | 56 (54) | 66.39 | 21.20 | 59.33 | 22.79 | -7.67 | 21.28 | -11.55% | **0.011** |
|  | 70 - 89 years | 38 (38) | 61.46 | 17.48 | 55.21 | 21.28 | -6.25 | 23.79 | -10.17% | 0.114 |
| **Body Mass Index** | Underweight | 14 (14) | 52.07 | 23.77 | 48.00 | 22.95 | -4.07 | 23.93 | -7.82% | 0.535 |
|  | Normal weight | 171 (165) | 66.33 | 18.96 | 58.98 | 20.68 | -6.97 | 18.89 | -10.51% | **<0.001** |
|  | Overweight | 114 (113) | 64.71 | 20.83 | 61.27 | 20.01 | -3.39 | 22.60 | -5.24% | 0.114 |
|  | Obese I | 40 (38) | 59.63 | 19.54 | 52.66 | 21.76 | -7.47 | 22.03 | -12.53% | **0.043** |
|  | Obese II | 5 (5) | 67.60 | 17.29 | 69.00 | 17.46 | 1.40 | 8.65 | 2.07% | 0.736 |
| **Age of Onset** | Below 40 years | 172 (168) | 63.53 | 20.84 | 56.99 | 21.20 | -6.04 | 20.76 | -9.50% | **<0.001** |
|  | Above 40 years | 172 (167) | 65.37 | 19.16 | 60.48 | 20.35 | -5.11 | 20.62 | -7.82% | **0.002** |
| **SCA type** | SCA 1, 2, 3 | 279 (270) | 64.75 | 20.22 | 58.57 | 20.83 | -6.00 | 20.20 | -9.27% | **<0.001** |
|  | SCA 6 | 65 (65) | 63.19 | 19.21 | 59.40 | 20.94 | -3.79 | 22.58 | -6.00% | 0.180 |
| **SARA Sum Score** | Mild ataxia severity | 105 (105) | 73.30 | 16.76 | 66.48 | 17.59 | -6.83 | 17.69 | -9.32% | **<0.001** |
|  | Moderate ataxia severity | 186 (181) | 62.49 | 18.88 | 56.33 | 20.52 | -5.95 | 21.26 | -9.53% | **<0.001** |
|  | Severe ataxia severity | 41 (37) | 54.66 | 24.13 | 52.92 | 24.13 | -0.08 | 21.92 | -0.15% | 0.982 |
|  | Extreme ataxia severity | 12 (12) | 50.83 | 18.55 | 45.00 | 21.43 | -5.83 | 30.38 | -11.48% | 0.520 |
| **INAS Count** | Less than 4 symptoms | 163 (160) | 67.55 | 19.11 | 62.66 | 19.23 | -4.85 | 19.02 | -7.18% | **0.001** |
|  | More than 4 symptoms | 181 (175) | 61.66 | 20.44 | 55.14 | 21.62 | -6.24 | 22.10 | -10.12% | **<0.001** |
| **PHQ-9 Sum Score** | No depression | 172 (169) | 70.28 | 17.54 | 65.67 | 17.78 | -4.49 | 19.81 | -6.39% | **0.004** |
|  | Mild depression | 102 (100) | 64.37 | 18.06 | 55.70 | 20.07 | -8.66 | 19.34 | -13.45% | **<0.001** |
|  | Moderate depression | 33 (32) | 55.03 | 21.73 | 47.03 | 19.91 | -6.91 | 20.49 | -12.55% | 0.066 |
|  | Severe depression | 37 (34) | 46.00 | 20.64 | 44.12 | 23.91 | -0.65 | 27.32 | -1.41% | 0.891 |

**Supplementary Table 6.** Multivariate linear regression model analysing factors influencing EQ VAS from baseline to each follow-up time point using differences in outcome variables (deltas) (n=344)

|  | **Baseline to 1-year Follow-up^1^ (n=330)** | | **Baseline to 2-year Follow-up^2^ (n=336)** | | **Baseline to 3-year Follow-up^3^ (n=335)** | |
| --- | --- | --- | --- | --- | --- | --- |
|  | ß (SE) | CI^95^ | ß (SE) | CI^95^ | ß (SE) | CI^95^ |
| Age | 0.162 (0.186) | [-0.204; 0.529] | -0.209** (0.177) | [-0.558;-0.140] | **-0.370* (0.177)** | **[-0.719;0.021]** |
| BMI | **-0.747** (0.264)** | **[0.228;1.266]** | 0.243 (0.253) | [-0.254;0.741] | 0.115 (0.249) | [-0.374;0.604] |
| Female gender (reference male) | **-4.600* (2.124)** | **[-8.779;-0.4212]** | -1.654 (2.015) | [-5.618;2.309] | 1.744 (1.964) | [-2.121;5.608] |
| Age of Onset | 0.048(0.206) | [-0.357;0.453] | -0.224 (0.199) | [-0.168;0.616] | 0.310 (0.199) | [-0.081;0.700] |
| Change of SARA between visits | **-0.737^┼^ (0.401)** | [-1.526; 0.051] | **-0.935** (0.350)** | [-1.624;-0.246] | **-0.859** (0.298)** | **[-1.444;-0.273]** |
| Change of INAS between visits | 0.416 (0.660) | [-0.882;1.715] | -0.712 (0.548) | [-1.790;0.365] | -0.124 (0.507) | [-1.122;0.873] |
| Change of PHQ-9 between visits | **-0.989*** (0.240)** | [-1.461;-0.516] | **-0.477* (0.213)** | [-0.897;-0.058] | **-0.493* (0.204)** | **[-0.895;-0.091]** |
| SCA type 6  (ref. SCA 1, 2, 3) | 0.309 (3.306) | [-6.196;6.813] | -4.904 (3.129) | [-11.060;1.251] | 0.676 (3.065) | [-5.354;6.706] |
| RLS syndrome | -2.936 (4.835) | [-12.449;6.577] | -0.232 (4.653) | [-9.386;8.921] | **-2.503* (4.530)** | **[-11.414;6.408]** |

**^┼^,** *, **, *** - levels of significance (<0.1, <0.05, <0.01, <0.001); ^1^ R^2^ 0.301; Adjusted R^2^ 0.279; p = <0.001 ; ^2^ R^2^ 0.211; Adjusted R^2^ 0.187; p = <0.001 ; ^3^ R^2^ 0.277; Adjusted R^2^ 0.255; p = <0.001; due to the lack of EQ VAS values at baseline not all values were imputed resulting in differing EQ VAS values at differing time points

| **Supplementary Table 7.** Change of EQ-5D-3L-levels from baseline to 3-year follow-up (n=344) | | | | | |
| --- | --- | --- | --- | --- | --- |
|  |  | **Baseline** | **1-year follow-up** | **2-year follow-up** | **3-year follow-up** |
|  |  | n (%) | n (%) | n (%) | n (%) |
| **Mobility** | **1** | 51 (14.83%) | 57 (16.86%) | 56 (16.42%) | 45 (13.08%) |
|  | **2** | 284 (82.56%) | 274 (81.07%) | 275 (80.65%) | 289 (84.01%) |
|  | **3** | 9 (2.62%) | 7 (2.07%) | 10 (2.93%) | 10 (2.91%) |
| **Self-care** | **1** | 242 (70.35%) | 206 (60.95%) | 187 (54.84%) | 183 (53.2%) |
|  | **2** | 91 (26.45%) | 118 (34.91%) | 131 (38.42%) | 143 (41.57%) |
|  | **3** | 11 (3.2%) | 14 (4.14%) | 23 (6.74%) | 18 (5.23%) |
| **Usual Activities** | **1** | 133 (38.66%) | 122 (36.09%) | 108 (31.67%) | 108 (31.4%) |
|  | **2** | 178 (51.74%) | 182 (53.85%) | 196 (57.48%) | 203 (59.01%) |
|  | **3** | 33 (9.59%) | 34 (10.06%) | 37 (10.85%) | 33 (9.59%) |
| **Pain/**  **Discomfort** | **1** | 179 (52.03%) | 175 (51.78%) | 177 (51.91%) | 178 (51.74%) |
|  | **2** | 153 (44.48%) | 149 (44.08%) | 148 (43.4%) | 148 (43.02%) |
|  | **3** | 12 (3.49%) | 14 (4.14%) | 16 (4.69%) | 18 (5.23%) |
| **Anxiety/ Depression** | **1** | 199 (57.85%) | 189 (55.92%) | 181 (53.08%) | 174 (50.58%) |
|  | **2** | 127 (36.92%) | 134 (39.64%) | 137 (40.18%) | 150 (43.6%) |
|  | **3** | 18 (5.23%) | 15 (4.44%) | 23 (6.74%) | 20 (5.81%) |
| **Total** |  | 344 | 341 | 338 | 344 |

**Members of the ESMI Study group:**

Marcus Grobe-Einsler, Arian Taheri Amin, Tamara Schaprian, Alhassane Diallo, Sophie Tezenas du Montcel

**Members of the EUROSCA Study group:**

Jeanette Hübener-Schmid, Kirsten Kuhlbrodt, Julien Peladan, Magda M. Santana, Holger Hengel, Kathrin Reetz, Hector Garcia-Moreno, Mafalda Raposo, Judith van Gaalen, Jon Infante, Katharina M. Steiner, Jeroen de Vries, Marcel M. Verbeek, Paola Giunti, Luis Pereira de Almeida, Manuela Lima, Bart van de Warrenburg, Ludger Schöls, Matthis Synofzik
